# Clinical Challenges with Talimogene Laherparepvec: Cured Lymph Nodes Masquerading as Active Melanoma

**DOI:** 10.1155/2019/4683531

**Published:** 2019-03-07

**Authors:** Umang Swami, Brian Swick, Yousef Zakharia, Mohammed Milhem

**Affiliations:** ^1^Department of Hematology, Oncology and Blood and Marrow Transplantation, University of Iowa Hospitals and Clinics, 200 Hawkins Dr, Iowa City, IA 52242, USA; ^2^Department of Dermatology and Pathology, University of Iowa Hospitals and Clinics, 200 Hawkins Dr, Iowa City, IA 52242, USA

## Abstract

Talimogene laherparepvec is a novel, genetically engineered, oncolytic herpes virus approved for local treatment of unresectable cutaneous, subcutaneous, and nodal lesions in patients with melanoma recurrent after initial surgery. It is administered as an intralesional injection. However, if the lesion continues to persist, it presents with a clinical challenge as when to stop treatment. Herein, we present two cases from our institution wherein the disease appeared to be persistent radiologically; however, on pathological excision, there was no evidence of disease and patients continue to be in durable remission after stopping treatment.

## 1. Introduction

Talimogene laherparepvec is a first-in-class, recombinant, intralesional, oncolytic virus therapy which has been approved by the United States Food and Drug Administration for local treatment of unresectable cutaneous, subcutaneous, and nodal lesions in patients with melanoma recurrent after the initial surgery [1]. It is a genetically modified herpes simplex virus type 1, in which infected cell protein (ICP) 34.5 is deleted which suppresses viral pathogenesis and enhances preferential viral replication in cancer cells. There is also translocation of the US11 gene following the *α*47 promoter (that regulates expression of ICP47) which causes its expression from a late gene to immediate early gene, enhancing virus replication and oncolysis. In addition, ICP47 gene is also deleted, and the gene for human granulocyte macrophage colony-stimulating factor (GM-CSF) is inserted to improve antigen presentation and T-cell priming [1, 2]. In a randomized phase III OPTiM trial, administration of intralesional talimogene laherparepvec significantly improved durable response rate (defined as response lasting at least 6 months continuously and beginning in the first 12 months of treatment) as compared to subcutaneous GM-CSF (16 vs. 2%, odds ratio 8.9; *p* < 0.001) [3]. However, it has not been shown to improve overall survival or to affect visceral metastasis [4].

Herein, we present two clinically challenging cases of patients undergoing talimogene laherparepvec-based treatment, where radiologically the disease appeared to be persistent even though pathologically the tumor was absent.

## 2. Case Presentations

### 2.1. Case 1

A 50-year-old Caucasian male with no significant past medical history underwent biopsy of a left flank lesion. Pathology revealed malignant melanoma, nodular type with 3.37 mm Breslow depth, Clark's level IV, nonulcerated, and mitotic grade of 4/mm^2^. PET/CT did not reveal metastatic disease. He underwent wide local excision with no residual melanoma. Two sentinel lymph nodes from the left axilla and left inguinal region were biopsied of which left inguinal lymph node showed microscopic foci of metastatic melanoma. Thereafter, he underwent left inguinal lymphadenectomy. Overall, 14 lymph nodes were dissected, and no melanoma was identified. Adjuvant interferon was tried, but he could not tolerate it. He thereafter continued to follow-up with surveillance imaging. Three and a half years later PET/CT revealed uptake in the right inguinal region. An ultrasound-guided fine needle aspiration revealed metastatic melanoma of the right inguinal lymph node bed. There was no evidence of any other site of metastasis, and the patient was determined to be stage IV (T3a, N1a, and M1a) melanoma. He started treatment on a clinical trial of talimogene laherparepvec with ipilimumab (NCT01740297). His Eastern Cooperative Oncology Group Performance Status (ECOG PS) was 0.

**Figure 1:**
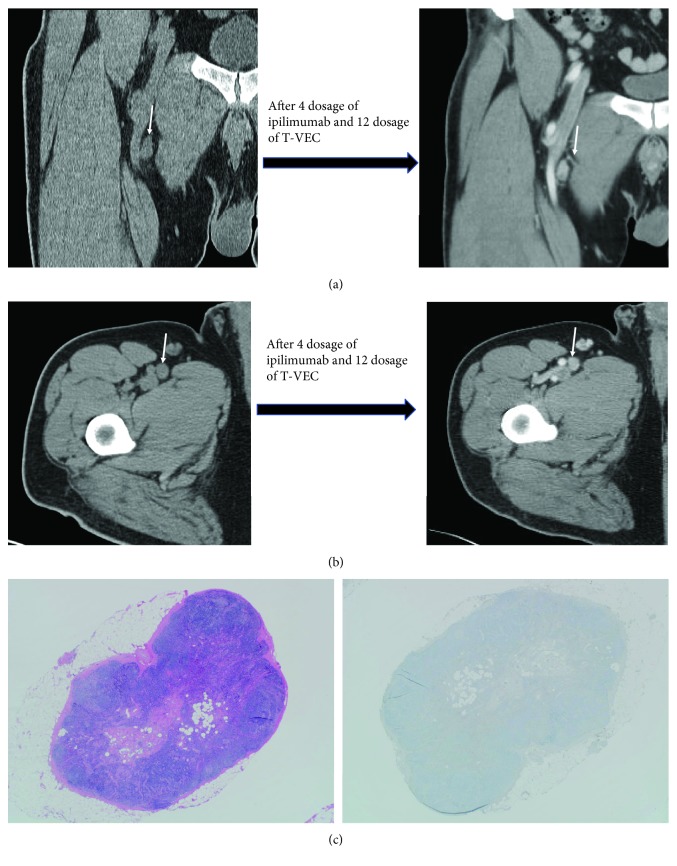
(a) Case 1 CT scan images at baseline and after treatment revealing persistent lymph nodes (marked by arrow) of similar size. (b) Case 1 CT scan images at baseline and after treatment revealing persistent lymph nodes (marked by arrow) of similar size. (c) Normal lymph node with no metastatic tumor present (hematoxylin and eosin stain and Mart-1-stained sections, 20x magnification).

In this phase Ib/II study, talimogene laherparepvec was administered intratumorally in week 1 (10(6) plaque-forming units/mL), then in week 4 and every 2 weeks thereafter (10(8) plaque-forming units/mL) along with ipilimumab (3 mg/kg) administered intravenously every 3 weeks for four dosages, beginning week 6 [5, 6]. The patient experienced fatigue, fever, chills, rigors, pruritus, rash, headaches, blurry vision, and abdominal discomfort (all grade 1) during treatment. Four months into the trial and after 2 months of finishing ipilimumab, the patient continued to show persistent right inguinal lymph nodes with no evidence of disease progression (Figures 1(a) and 1(b)). A fine needle aspiration revealed only reactive lymph nodes. A decision was made to perform a limited right femoral lymphadenectomy. Pathology review of all excised lymph nodes did not reveal any evidence of melanoma (0/5 Figure 1(c)). He did not develop any significant complications after lymphadenectomy. The patient was on active surveillance after lymph node dissection and continues to be in remission for the last 5 years without any subsequent treatment.

### 2.2. Case 2

**Figure 2:**
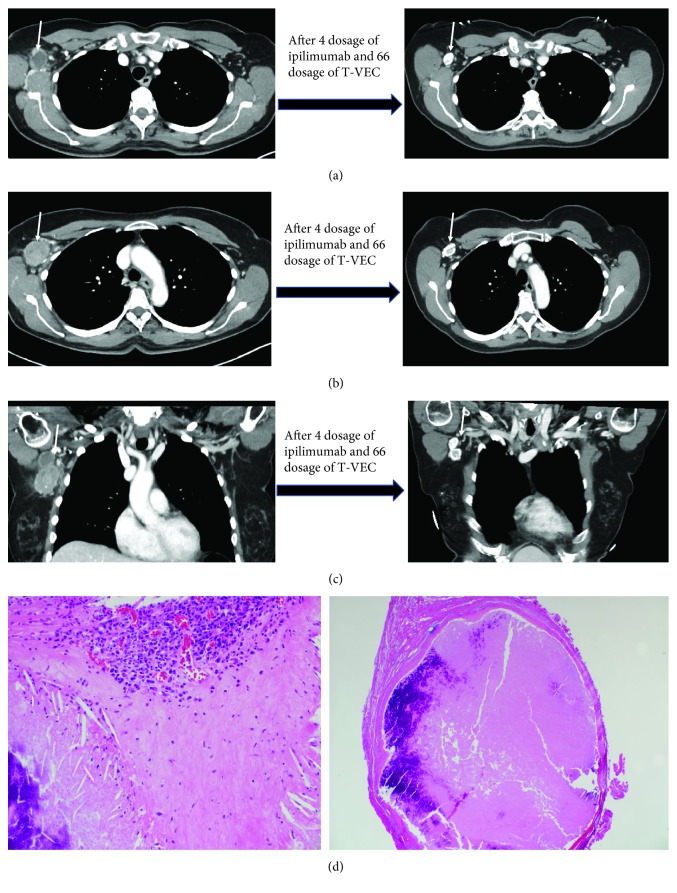
(a) CT images of case 2 showing persistent lymph nodes (marked by arrow) with decreased size and calcification. (b) CT images of case 2 showing persistent lymph nodes (marked by arrow) with decreased size and calcification. (c) CT images of case 2 showing persistent lymph nodes (marked by arrow) with decreased size and calcification. (d) Necrotic lymph node with infiltrating inflammatory cells and no viable tumor present (hematoxylin- and eosin-stained sections, 20x and 100x magnification).

A 57-year-old female with no significant comorbidities was diagnosed with melanoma of right upper back after a biopsy. Pathology revealed Clark's level IV, Breslow thickness 0.87 mm superficial spreading melanoma with no ulceration, and mitosis rate of 1/mm^2^. She underwent wide local excision with no residual melanoma. No sentinel lymph node biopsy was done. She was followed by active surveillance without evidence of disease, until approximately 5 years later when she had a palpable right axillary mass, biopsy of which confirmed metastatic melanoma. PET/CT and MRI brain did not reveal any other metastatic sites, and she was determined to be as stage IIIC (pT1b, pN2b, and cM0) melanoma. The patient started treatment on a clinical trial of talimogene laherparepvec with ipilimumab (NCT01740297) [5, 6]. Her ECOG PS was 0. The patient experienced right axillary and shoulder pain and burning, fatigue, and nausea (all grade 1). Two and a half years into the trial, the patient experienced partial response with persistent evidence of lymph nodes on CT scans (Figures 2(a)–2(c)). A decision was made to perform adjuvant right axillary lymph node dissection after the patient had undergone 66 talimogene laherparepvec injections. Pathology review of 11 dissected lymph nodes did not show any evidence of melanoma (Figure 2(d)). After surgery, she developed right breast lymphedema, but no lymphedema in the right upper arm. The patient continues to be in remission for the last 8 months.

## 3. Discussion

Cancer immunotherapies have demonstrated different patterns of responses ranging from pseudoprogression to hyperprogression and dissociated responses [7]. Pseudoprogression, which is likely caused by acute (antitumoral) inflammation, can lead to a false indication that the treatment is not working leading to premature discontinuation of therapy [7, 8] while “pseudolatency” (inflammation) may lead to continuation of treatment beyond requirement and may arise from chronic exposure to immunotherapeutic agents. Both phenomena present clinical challenges with regard to treatment planning. To help with these many approaches are being investigated. These include imaging techniques like FDG-PET as eloquently described by Koski et al. [9] and novel biomarkers like serum high-mobility group box 1 (HMGB1) protein, immunoglobulin-like transcript 2 (ILT2), and IL-8 [10–12]. Serum HMGB1 is a predictive and prognostic biomarker for oncolytic immunotherapy with adenovirus [10] while IL-8 appears to be a promising candidate for adenoviral immunotherapy [12]. ILT2 has shown to be a biomarker of therapeutic response to oncolytic vaccinia virus immunotherapy [11].

The exact mechanism of action of talimogene laherparepvec is unknown [4]. It is believed to use cell-surface-bound nectins to enter cancer cells and then selectively replicate within them by disrupting protein kinase R (PKR) activity and type I interferon signaling [2]. After viral replication, propagation, and assembly, talimogene laherparepvec triggers oncolysis leading to release of tumor-derived antigens which along with virally derived GM-CSF, viral-based pathogen-associated molecular pattern factors, cell-derived danger-associated molecular pattern molecules, interferons, and other cytokines which recruit and facilitate maturation of antigen-presenting cells lead to presentation of tumor-associated antigens to cytotoxic CD8+ T cells which subsequently promote antitumor response [1, 2, 4]. At the same time, new released viral particles infect surrounding tumor cells and propagate the treatment [1, 2]. Talimogene laherparepvec has been investigated in combination with other agents including pembrolizumab as well as chemotherapy with radiation in clinical trials [13]. It has also recently demonstrated promising activity with MEK inhibitors in preclinical melanoma models [14]. The current status of oncolytic virotherapy in combination cancer immunotherapy has been extensively reviewed by Bommareddy et al. [13].

Though talimogene laherparepvec is a novel therapy, it brings clinical challenges with regard to when to stop treatment. As discussed above in cases 1 and 2, it was very hard to determine whether melanoma in injected lesions has undergone remission or residual disease is still present. In this scenario, PET/CT will show an FDG avid node due to chronic inflammation because of repeated injections. Performing a biopsy always comes with a risk of missing the disease and a negative biopsy will thus not convincingly rule out the persistent disease. Stopping treatment may come with the risk of disease spread. However, continuing with treatment leads to financial toxicity, treatment-related side effects, and anxiety related to treatment and disease. Therefore, complete excision of the lesion or lymph node dissection as in our cases remains the sole alternative to determine whether a patient is in complete remission or not. Therefore, there is a need for a novel biomarker to help determine whether it is advisable to stop talimogene laherparepvec injections or to continue it in these challenging clinical scenarios.
